# A theory-based study of doctors’ intentions to engage in professional behaviours

**DOI:** 10.1186/s12909-020-1961-8

**Published:** 2020-02-10

**Authors:** Antonia Rich, Asta Medisauskaite, Henry W. W. Potts, Ann Griffin

**Affiliations:** 1 Research Department of Medical Education, UCL Medical School, Room GF/664, Royal Free Hospital, London, NW3 2PF UK; 2 UCL Institute of Health Informatics, 222 Euston Road, London, NW1 2DA UK; 3 Research Department of Medical Education, UCL Medical School, 74 Huntley Street, London, WC1E 6AU UK

## Abstract

**Background:**

The Theory of Planned Behaviour (TPB) has been proposed as an appropriate model for creating a theory-driven approach to teaching medical professionalism. However, there is a lack of empirical evidence into its efficacy. This study explores if the TPB can assess UK medical doctors’ professional behaviours and explores if there are differences in the TPB’s efficacy depending on doctors’ primary medical qualification (UK or outside).

**Methods:**

Three hundred fourteen doctors in England at 21 NHS Trusts completed a questionnaire about reflective practice, using the General Medical Council’s confidentiality guidance, and raising a patient safety concern. The majority of participants were male (52%), white (68%), consultants (62%), and UK medical graduates (UKGs) (71%).

**Results:**

The TPB variables of attitudes, subjective norms, and perceived behavioural control were predictive of intention to engage in raising concerns (R^2^ = 35%), reflection (R^2^ = 52%), and use of confidentiality guidance (R^2^ = 45%). Perceived behavioural control was the strongest predictor of intentions to raise a concern (β = 0.44), while attitude was the strongest predictor of intentions to engage in reflective practice (β = 0.61) and using confidentiality guidance (β = 0.38). The TBP constructs predicted intention for raising concerns and reflecting for both UKGs and non-UKGs (*F*s ≥ 2.3; *p*s ≤ .023, βs ≥ 0.12). However, only perceived behaviour control was predictive of intentions to use guidance for both UKGs and non-UKGs (β = 0.24) while attitudes and norms were just predictive for UKGs (βs ≥ 0.26).

**Conclusions:**

This study demonstrates the efficacy of the TPB for three professional behaviours. The implications for medical educators are to use the variables of the TPB (attitudes, subjective norms, and perceived behavioural control) in the education of professionalism, and for medical education researchers to further our understanding by employing the TPB in more empirical studies of non-clinical behaviours.

## Background

Despite medical organisations around the world highlighting the need for increased emphasis on professionalism in medical education [1], there is no widely accepted definition of professionalism [2] and no unifying theoretical model that guides the integration of professionalism into medical education [3, 4]. There has been growing recognition of the need for theory-based research to understand healthcare professionals’ behaviours, and to inform the design of interventions intended to change these behaviours [5–8]. Archer and colleagues [4] propose that the Theory of Planned Behaviour (TPB) would be an appropriate model for creating a more unified, theory-driven approach to teaching medical professionalism and that future research should investigate the variables of the TPB, i.e. attitudes, subjective norms and perceived behavioural control, on professionalism. Other authors have also suggested the TPB as a useful framework to evaluate professionalism [9]. However, while the TPB has been proposed as an appropriate theory for integrating professionalism training in medical education, there is a lack of empirical evidence that examines its efficacy. Therefore, this study aims to examine the utility of the TPB for predicting doctors’ professional behaviours: specifically, raising a patient safety concern, carrying out reflective practice, and using the General Medical Council’s (GMC’s, responsible for the regulation of doctors in the UK) guidance on confidentiality.

**Fig. 1:**
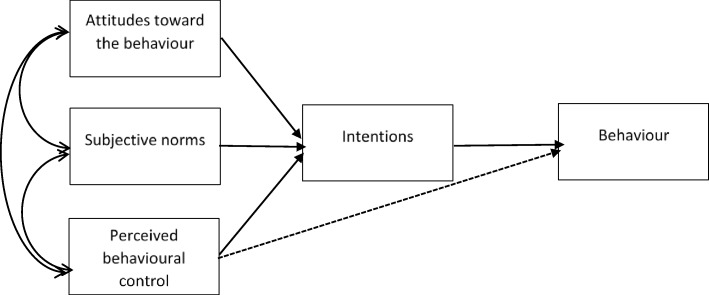
Theory of Planned Behaviour

Systematic reviews examining the Theory of Reasoned Action (TRA) [10] and its extension, the TPB [11], have concluded that the theories are able to predict intentions and behaviours among different groups of clinicians, including doctors [7, 12, 13]. According to the TPB (Fig. 1), intentions are the precursor of behaviours, and the stronger the intention, the more likely the behaviour is to be performed. Intention is determined by three variables: 1) attitudes (overall evaluation of the behaviour), 2) subjective norms (estimation of the social pressure to carry out the behaviour), and 3) perceived behavioural control (the extent to which a person feels able to perform the behaviour). Given the challenges of measuring actual behaviour, intention can be used as a proxy, where a positive relationship between intention and behaviour has been confirmed [14]. This assumption has been supported for behaviours among clinicians [5].

The TPB has been frequently applied to understand clinicians’ behaviours. For example, in a systematic review examining healthcare professionals’ intentions and behaviours using social cognitive theories, Godin and colleagues found that the theory used most frequently was the TRA or TPB, which was able to explain approximately 35% of the variance in behaviours and 59% of the variance in intentions [7]. However, the behaviours were mainly performed within a clinical context (e.g., prescribing or adhering to clinical guidelines). While the TPB has also been used to study other type of behaviours, such as ethical decision making (reporting a medical error) [15], there is a paucity of studies using the TPB to examine doctors’ professionalism. Understanding the factors that influence doctors’ intentions to raise a patient safety issue, reflect on their practice, and use professional guidance is critical to improving patient safety [16].

Professionalism in this paper is defined as consisting of three professional behaviours: raising a patient safety concern, engaging in reflective practice, and using confidentiality guidance. Doctors have a professional duty to act if they have a concern about patient safety [17], to regularly reflect on their standards of practice, and to abide by guidance in confidentiality [18]. However, in real world practice, all three behaviours can be challenging to perform. For example, research has identified multiple barriers to speaking about patient safety concerns, such as organisational culture norms, power dynamics, and fears of damaging relationships [19–23]. Similarly, reflection is a complex construct which does not have a singular agreed definition and comes with its own challenges. The recent case of Dr. Bawa-Gaba, the trainee paediatrician convicted of medical negligence and removed from the UK medical register following the death of a child until winning an appeal, sparked much controversy regarding reflective practice [24]. This led to many doctors feeling they are no longer able to reflect honestly, openly and safely, due to fears of recrimination [25]. Confidentiality is fundamental to doctors’ professionalism and of great importance to patients [26, 27]; while research has shown that doctors’ attitudes to confidentiality guidance is generally positive, organisational norms and a lack of resources can mean confidentiality is unintentionally breached [28].

Consistent with the TPB, we hypothesize that doctors’ attitudes towards the behaviour, subjective norms, and their perceived behavioural control will predict intentions to engage in professional behaviours. It is, however, important to consider differences between groups of doctors. Healthcare provision relies on non-UK graduates (non-UKGs) [29] who account for a significant proportion of the National Health Service (NHS) workforce [30], but who are more likely to face fitness to practise investigation [31]. Studies show that UK and non-UKGs’ attitudes toward professional behaviours, as well as engagement in these behaviours, differ. Non-UKGs were more likely to have referred to GMC guidance over the past 12 months than UKGs (63% vs. 50%), while UKGs were more likely to state they had concerns for patient safety (17% of UKGs; 11% of International Medical Graduates (IMG); 15% European Economic Area (EEA)) [32]. Understanding what factors link to doctors’, especially non-UKGs’, engagement in professional behaviours will help to develop more appropriate interventions targeting this particular group of doctors.

Thus, the aim of this study is (i) to examine whether the TPB has utility for understanding doctors’ professional behaviour in three areas (raising concerns, engaging in reflective practice, and using confidentiality guidance); and (ii) whether there are differences between UK and non-UK graduates.

## Methods

### Context of the study

The study reported in this article uses data collected at the baseline of non-randomised experimental research. The larger research is a GMC funded study to investigate the effectiveness of the “Duties of a Doctor” (DoaD) programme, the GMC’s programme of preventative educational workshops.

### Development of the questionnaire

The questionnaire was designed based on published guidance for constructing a TPB questionnaire [33] and was piloted with eight doctors to ascertain practical aspects (e.g., timings) and face validity, which led to minor changes in wording and formatting.

The final questionnaire included demographic questions (e.g., gender, role, work experience) and 73 items about three professional behaviours in four TPB dimensions. The TPB dimensions were:

1. **Attitudes.** The doctor’s overall evaluation of the behaviour.
2. **Subjective norms**. The degree of pressure felt from various organisations and people to act in a certain way (e.g., peers).
3. **Perceived behaviour control**. Doctors’ confidence and beliefs about their ability to carry out the behaviour.
4. **Intentions**. The extent to which doctors’ intend to carry out the behaviour in the future.

Attitudes, subjective norms, perceived behavioural control, and intentions were measured on a 7-point bipolar or Likert scale scored from 1 to 7. Higher scores showed more positive attitudes, norms, perceived control, and intentions. Cronbach’s α was calculated for each of the four TPB variables for the three professional behaviours. To improve internal consistency reliability (if lower than .6) items which were poorly correlated with others in the scale were eliminated.

The three professional behaviours were (see questionnaire description in Additional file 11: Table S1):

1. **Raising concerns.** 23 items measured raising concerns, but two items from the attitude scale were excluded from the analysis as they negatively correlated with the other items (Raising a concern is: “*the wrong thing to do – the right thing to do*”; “*bad practice – best practice*”). One item from the perceived behavioural control scale (*Whether I report a patient safety concern is entirely up to me*) was excluded to improve internal consistency (initial Cronbach’s α = .32). The final scales: attitudes (4 items; e.g., *Overall, I think that raising a concern is worthless-worthwhile*), subjective norms (11 items; *It is expected of me that I report a concern if I have one*), perceived behaviour control (2 items; *I am confident that I can raise*), intentions (3 items; *I plan to raise a concern if I have one in my work place*).
2. **Reflection**. 24 items measured reflective practice, but two items were excluded from the analysis from the perceived behaviour control scale (*I am confident that I cannot reflect on my practice; Whether I reflect on my practice is entirely up to me*) to improve internal consistency (initial Cronbach’s α = −.12). The final scales: attitudes (8 items; *Reflecting on my practice makes me a better doctors*), subjective norms (12 items; *People who are important to me think I should reflect on my practice*), perceived behaviour control (1 item; *For me to reflect on my practice is difficult-easy*), intentions (3 items; *I intend to reflect on my practice*).
3. **Use of confidentiality guidance**. 27 items measured use of confidentiality guidance, but one item was excluded from the analysis from the perceived behaviour control scale (*Whether I use the GMC confidentiality guidance is entirely up to me*) to improve internal consistency (initial Cronbach’s α = .45). The final scales: attitudes (8 items; *Overall, I think GMC confidentiality guidance is Unrealistic-Realistic*), subjective norms (11 items; *It is expected of me to use the GMC confidentiality guidance*), perceived behaviour control (4 items; *I have enough time to refer to the GMC confidentiality guidance*), intentions (3 items; *I intend to refer to the GMC confidentiality guidance the next time I’m uncertain*).

### Procedure

Data were collected via paper and online questionnaires between September 2017 and March 2018, at 21 NHS Trusts and surgeries in England. Doctors attending the DoaD programmes received an invitation via email to take part in this study prior to the first session. The email included a link to the survey (Online survey platform). These doctors also had an option to fill in a paper-based questionnaire prior to starting the DoaD programme. The comparator group of doctors, not attending the programme, were invited to participate via email and completed the questionnaire online. These doctors were from the same Trusts as doctors taking part in the DoaD programme. The University College London (UCL) Research Ethics Committee approved this study (5490/001).

### Statistical analysis

Statistical analyses were performed with SPSS v24 [34]. All scales were approximately Normally distributed (skewness and kurtosis between − 2 and 2) [35]. Correlations were calculated with Pearson correlation coefficients, independent sample Student’s t-tests were used to compare mean scores between groups of doctors, and multiple linear regressions to examine the TPB. To explore an interaction between primary medical qualification (PMQ) and TBP factors, continuous variables were centred to avoid multicollinearity (all values between 1 and 3). Participants with missing values were excluded on an analysis by analysis basis.

## Results

### Participants

Three hundred fourteen doctors took part in the study. Demographic characteristics are presented and compared to the List of Registered Medical Practitioners (LRMP [36]) in Table 1. The LRMP contains demographic details of all doctors registered to practice in the UK and thus enables comparison of the representativeness of the sample. The majority of participants were male (52%), white (68%), consultants (62%), and UK graduates (71%). The largest group had more than 21 years of experience working as a doctor (40%). Demographic characteristics of doctors in this study were broadly similar to the LRMP, except a much higher proportion of doctors of consultant grade participated in this study compared to the LRMP.

**Table 1:** Participants’ demographic characteristics

Demographic characteristics		*n* (%)	LRMP
Gender			
Male		162 (51.6%)	54.5%
Female		147 (46.8%)	45.5%
Missing		5 (1.6%)	
Ethnic group			
White		213 (67.8%)	52.4%
BME		95 (30.3%)	31.8%
Prefer not to tell/Missing		6 (1.9%)	15.8%
PMQ			
UK		217 (70.9%)	63%
Non-UK		89 (29.1%)	37%
Role			
Consultant		196 (62.4%)	31.6%
Trainee on an HEE training programme		15 (4.8%)	21%
Foundation stage		27 (8.6%)	
General practitioner		13 (4.1%)	23.5%
Other (e.g., Staff Grade, Associate Specialist, Trust Grade, etc.)		63 (20.1%)	23.3%
Experience (years)			
< 1		16 (5.1%)	n/a
1–4		45 (14.3%)	n/a
5–10		28 (8.9%)	n/a
11–20		99 (31.5%)	n/a
> 21		124 (39.5%)	n/a
Missing		2 (0.6%)	n/a

*Note. LRMP* the List of Registered Medical Practitioners,*BME* Black and Minority Ethnic, *PMQ* Primary Medical Qualification, *UK* United Kingdom, *HEE *Higher Education England

### The utility of the TPB for predicting intentions to engage in professional behaviours

Table 2 reports correlation coefficients between the scales used in this study. The four TPB constructs related to the same professional behaviour significantly correlated with each other. Table 3 reports multiple linear regression analyses showing that the TPB constructs significantly predict intentions (*F*s ≥ 55.3; *p*s < .001) to engage in reflective practice (R^2^ = 52%), use confidentiality guidance (R^2^ = 45%), and raise concerns (R^2^ = 35%). The strongest predictor of intentions to raise concerns was perceived behaviour control (β = 0.44). Attitudes was the strongest predictor for both intentions to reflect (β = 0.61) and use confidentiality guidance (β = 0.38).

**Table 2:** Correlation matrix of attitudes, subjective norms, perceived behaviour control and intentions to raise concerns, reflect and use confidentiality guidance

Scale		Cronbach’s α	*M* (SD)	Raising concerns				Reflection				Use of confidentiality guidance			
				ATT	SN	PBC	INT	ATT	SN	PBC	INT	ATT	SN	PBC	INT
Raising concerns	ATT	0.67	3.99 (1.08)	1											
	SN	0.85	4.73 (1.08)	.126*	1										
	PBC	0.61	5.25 (1.28)	.507^**^	.199^**^	1									
	INT	0.69	5.64 (1.03)	.416^**^	.237^**^	.556^**^	1								
Reflection	ATT	0.87	5.3 (1.1)	.251^**^	.147^**^	.243^**^	.237^**^	1							
	SN	0.88	4.45 (1.12)	.143^*^	.506^**^	.088	.075	.214^**^	1						
	PBC	n/a^a^	5.18 (1.5)	.183^**^	−.031	.254^**^	.183^**^	.530^**^	.145^*^	1					
	INT	0.82	6 (1.09)	.249^**^	.164^**^	.221^**^	.356^**^	.692^**^	.298^**^	.451^**^	1				
Use of confidentiality guidance	ATT	0.84	4.7 (1.08)	.328^**^	.088	.378^**^	.337^**^	.418^**^	.188^**^	.244^**^	.264^**^	1			
	SN	0.94	4.07 (1.45)	.242^**^	.463^**^	.116^*^	.152^**^	.214^**^	.525^**^	.014	.166^**^	.300^**^	1		
	PBC	0.70	4.58 (1.1)	.269^**^	.169^**^	.341^**^	.367^**^	.325^**^	.204^**^	.205^**^	.268^**^	.672^**^	.330^**^	1	
	INT	0.83	5.17 (1.3)	.282^**^	.168^**^	.251^**^	.394^**^	.381^**^	.224^**^	.138^*^	.404^**^	.604^**^	.388^**^	.570^**^	1

*Note*. *ATT* Attitudes, *SN* Subjective norms, *PBC* Perceived behavioural control, *INT* Intentions; ^a^Scale consists of one item

**p < 0.01; *p < 0.05

**Table 3:** Regressions predicting intention to raise concerns, reflect and use confidentiality guidance from attitudes (ATT), subjective norms (SN) and perceived behaviour control (PBC)

		Unstandardized Coefficient	Standardised Coefficient	t	*P*	CI 95%	
		B	Beta			LB	UB
Raising concerns	Constant	2.51		9.0	**<.001**	1.96	3.06
	ATT	0.17	0.18	3.3	**.001**	0.07	0.27
	SN	0.12	0.13	2.8	**.006**	0.04	0.21
	PBC	0.36	0.44	8.2	**<.001**	0.27	0.44
	Model	*F*(3, 309) = 55.3; *p* < .001; R^2^ = 0.349					
Reflection	Constant	1.73		6.9	**<.001**	1.24	2.22
	ATT	0.61	0.61	13.1	**<.001**	0.51	0.70
	SN	0.15	0.16	3.9	**<.001**	0.08	0.23
	PBC	0.07	0.10	2.2	**.028**	0.01	0.14
	Model	*F*(3, 307) = 112.8; *p* < .001; R^2^ = 0.524					
Use of confidentiality guidance	Constant	0.95		3.5	**.001**	0.42	1.49
	ATT	0.46	0.38	6.5	**<.001**	0.32	0.59
	SN	0.17	0.19	4.3	**<.001**	0.09	0.25
	PBC	0.30	0.26	4.4	**<.001**	0.17	0.44
	Model	*F*(3,308) = 78.4; *p* < .001; R^2^ = 0.447					

*Note*. *LB* Lower Bound, *UB* Upper Bound, *ATT* Attitudes, *SN* Subjective norms, *PBC* Perceived behavioural control, *INT* Intentions

### Differences between UKGs and non-UKGs

Table 4 presents the comparison of the TPB constructs between UKGs and non-UKGs in the three professional behaviours. Significant differences between groups of doctors were found analysing six TPB variables. Non-UKGs expressed significantly more positive attitudes towards raising concerns (*t*(303) = − 3.8; *p* < .001), reflective practice (*t*(304) = − 4.3; *p* < .001), and held stronger intentions to reflect (*t*(208.4) = − 2.8; *p* = .005). Regarding confidentiality guidance, they had more positive attitudes (*t*(304) = − 2.0; *p* = .048), stronger subjective norms (*t*(304) = − 2.3; *p* < .001), and greater intentions to use the guidance (*t*(224.3) = − 4.9; *p* < .001) compared to UKGs.

**Table 4:** Comparison of the TPB constructs among UK and non-UK graduates in three professional behaviours: a) raising concerns, b) reflective practice and c) use of confidentiality guidance)

Professional behaviour	TPB factor	PMQ	*M* (*SD*)	t-test statistics
Raising concerns	**ATT**	**UK**	**3.84 (1.03)**	***t*(303) = −3.8;** ***p*** **< .001**
		**Non-UK**	**4.35 (1.12)**	
	SN	UK	4.75 (1.01)	*t*(304) = 0.6; *p* = 553
		Non-UK	4.67 (1.20)	
	PBC	UK	5.19 (1.30)	*t*(304) = −1.5; *p* = .147
		Non-UK	5.42 (1.20)	
	INT	UK	5.59 (1.06)	*t*(303) = −1.5; *p* = .143
		Non-UK	5.78 (0.91)	
Reflective practice	**ATT**	**UK**	**5.13 (1.11)**	***t*(304) = −4.3;** ***p*** **< .001**
		**Non-UK**	**5.70 (0.95)**	
	SN	UK	4.40 (1.03)	*t*(304) = −0.6; *p* = .580
		Non-UK	4.49 (1.32)	
	PBC	UK	5.10 (1.54)	*t*(302) = −1.3; *p* = .209
		Non-UK	5.34 (1.40)	
	**INT**	**UK**	**5.89 (1.15)**	***t*(304) = −2.8;** ***p*** **= .005**
		**Non-UK**	**6.24 (0.89)**	
Use of confidentiality guidance	**ATT**	**UK**	**4.62 (1.06)**	***t*(304) = −2.0;** ***p*** **= .048**
		**Non-UK**	**4.89 (1.09)**	
	**SN**	**UK**	**3.96 (1.41)**	***t*(304) = −2.3;** ***p*** **< .001**
		**Non-UK**	**4.37 (1.53)**	
	PBC	UK	4.55 (1.08)	*t(*304) = −0.9; *p* = .390
		Non-UK	4.67 (1.12)	
	**INT**	**UK**	**4.97 (1.36)**	***t*(303) = −4.9;** ***p*** **< .001**
		**Non-UK**	**5.65 (0.97)**	

*Note*. *PMQ* Primary medical qualification, *ATT* Attitudes, *SN* Subjective norms, *PBC* Perceived behavioural control, *INT* Intentions

Table 5 presents results for multiple linear regressions: these included a term for place of primary medical qualification (PMQ, UKG vs. non-UKG) and all interactions between PMQ and TPB factors. All three models were significant (*F*s ≥ 23.4; *p*s < .001) explaining a substantial proportion of variance in intentions to raise concerns (36%), reflect (53%), and use confidentiality guidance (51%).

**Table 5:** Regressions predicting UK and non-UK graduate doctors’ intentions to engage in three professional behaviours

Professional behaviours	TPB factors	Unstandardized Coefficient	Standardised Coefficient	t	*p*	CI 95%		Model statistics
		B	Beta			LB	UB	
Raising concerns	Constant	2.30		7.5	**<.001**	1.69	2.91	*F*(7,297) = 23.4; *p* < .001; R^2^ = 0.356
	ATT	0.21	0.23	3.4	**.001**	0.09	0.34	
	SN	0.13	0.13	2.5	**.014**	0.03	0.23	
	PBC	0.36	0.45	7.2	**.000**	0.26	0.46	
	PMQ	0.06	0.02	0.5	.614	−0.16	0.27	
	ATT*PMQ	−0.11	−0.07	−0.9	.352	− 0.33	0.12	
	SN*PMQ	−0.12	−0.08	−1.6	.106	−0.26	0.03	
	PBC*PMQ	−0.06	−0.04	− 0.6	.582	− 0.25	0.14	
Reflective practice	Constant	1.31		4.5	**<.001**	0.74	1.88	*F*(7,296) = 48.3; *p* < .001; R^2^ = 0.533
	ATT	0.63	0.64	11.4	**<.001**	0.52	0.74	
	SN	0.20	0.20	3.7	**<.001**	0.09	0.30	
	PBC	0.09	0.12	2.3	**.023**	0.01	0.17	
	PMQ	0.05	0.02	0.5	.625	−0.15	0.25	
	ATT*PMQ	−0.14	−0.07	−1.2	.228	−0.36	0.09	
	SN*PMQ	−0.13	−0.09	−1.7	.097	−0.29	0.03	
	PBC*PMQ	−0.08	−0.05	−1.0	.330	−0.23	0.08	
Use of confidentiality guidance	Constant	0.25		0.8	.407	−0.35	0.85	*F*(7,297) = 44.6; *p* < .001; R^2^ = 0.513
	ATT	0.55	0.45	6.8	**<.001**	0.39	0.70	
	SN	0.23	0.26	4.8	**<.001**	0.14	0.32	
	PBC	0.28	0.24	3.8	**<.001**	0.13	0.44	
	PMQ	0.57	0.20	4.8	**<.001**	0.33	0.80	
	ATT*PMQ	−0.45	−0.20	−3.2	**.002**	−0.73	−0.17	
	SN*PMQ	−0.25	− 0.16	−3.1	**.002**	− 0.41	−0.09	
	PBC*PMQ	0.09	0.04	0.7	.513	−0.19	0.37	

*Note*. *ATT* Attitudes, *SN* Subjective norms, *PBC* Perceived behavioural control, *INT* Intentions, *PMQ* Primary medical qualification (0 = UK; 1 = non-UK); LB - Lower Bound; UB - Upper Bound

UKGs’ and non-UKGs’ intentions to raise concerns and reflect were not significantly different (*p*s ≥ .614) when controlling for TPB factors. More positive attitudes, stronger subjective norms and perceived behaviour control predicted stronger intentions to engage in these two professional behaviours in all of the sample (*F*s ≥ 2.3; *p*s ≤ .023, βs ≥ 0.12). The changes in intentions to raise concerns and reflect due to changes in the TPB factors did not differ between UKG and non-UKG (*p*s ≥ .097).

However, when it comes to the use of confidentiality guidance, there were significant differences by PMQ. Higher perceived behavioural control was predictive of higher intentions in the whole sample (β = 0.24, *p* < .001; interaction term not significant, *p* = .513). However, the effects of attitudes and subjective norms showed an interaction with PMQ whereby both were predictive of intentions in UKGs, but not in non-UKGs.

## Discussion

### TPB use in predicting professional behaviours

Understanding why doctors engage or not in professional behaviours is essential in order to promote good medical practice. This study empirically tested the utility of a theoretical model to investigate what factors contribute to such engagement. The findings demonstrated that the TPB had predictive efficacy to better understand doctor’s professionalism – raising concerns, carrying out reflective practice, and using confidentiality guidance. More positive attitudes, stronger subjective norms and greater perceived behavioural control significantly predicted stronger intentions to engage in these three behaviours.

The study results showed the TPB is able to explain between 35 and 52% of the variance in intentions, which represents a medium-to-large effect size, comparing favourably to other studies. For example, in a previous study the TPB constructs explained 32% of the variance in UK pharmacists’ intentions to report errors [37], which is similar to 35% we observed in the current study when analysing raising concerns. Likewise, 48% of the variance to use clinical guidance was explained among Finish doctors [38], compared to 45% to use confidentiality guidance in this study. It is not clear, however, why the variation in efficacy of prediction is observed between the different behaviours. Work environments and organisational factors might be more influential for some behaviours than others. A behaviour such as raising concerns may be more complex and dependent on other, non-cognitive, mechanisms (e.g., systems in place to act upon a concern) while a doctor has more control over his/her reflective practices and decision to consult regulator guidance. The decision to engage in reflective practice and use guidance is predominantly an individual behaviour, less reliant on external factors such as systems. In addition, both behaviours have arguably less potential negative implications than raising a concern does for a doctor, which has ramifications for others and come with a host of obstacles including organisational culture norms, hierarchies and power dynamics and anxiety about damaging relationships [19, 20, 22, 23].

### Differences between UKGs and non-UKGs

Previous studies recognised that non-UKGs are more likely to engage in professional behaviours, e.g., refer to the GMC for advice or use guidance [32]. The present study also revealed some differences between UK and non-UKGs’ intentions, with non-UKGs holding greater intentions to reflect and to use the guidance compared to UKGs. In addition, non-UKGs had more positive attitudes towards all three professional behaviours and had stronger subjective norms to engage in reflective practice.

Despite these differences, the study results showed that all three TPB factors have predictive utility for two professional behaviours (raising concerns, reflection) regardless of whether a doctor is a UK graduate or has obtained their primary medical qualification outside the UK. However, just perceived behavioural control was predictive of intentions to use guidance for both UKGs and non-UKGs, while attitudes and subjective norms predicted intentions to use guidance only for UKGs. It is perhaps because those who are trained outside the UK are less familiar with the guidance and, therefore, attitudes and subjective norms have less impact on their decision to consult guidance. Indeed, non-UKGs report that the ethical and legal frameworks in countries of their qualification differ a lot from the UK, where policies may be understood as much more legally based and prescriptive [39]. Such an approach creates a perception of the use of regulatory guidance as “a must” behaviour that may minimise the impact of cognitive mechanisms on intentions to use it. However, contrary to the use of regulator guidance, reflective practice and raising concerns are more fluid behaviours, perceptions of which are heavily affected by organisational climate (e.g. [40]) and, therefore, more influenced by attitudes and subjective norms for both, UKGs and non-UKGs.

### Strengths and limitations

The present study is a valuable contribution to the literature by demonstrating the applicability of the TPB to professional behaviours of doctors. The strengths of the study include the relatively large data set, which is broadly representative of doctors licensed to practice in the UK in terms of gender, ethnicity, and PMQ, although doctors of consultant grade were overrepresented.

One limitation is that the data is cross-sectional not longitudinal, and the measurement of intention as a proxy for behaviour. A more rigorous test of the TPB would have included a measurement of behaviour and used a prospective longitudinal design to examine the ability to predict future behaviour. There are few longitudinal studies of the assessment of professionalism and this warrants further research [41]. A second limitation is that we are unable to calculate a response rate for the questionnaire. This is due to a third party (i.e., NHS Trusts) disseminating emails to potential participants on our behalf and our attempts to gather precise data on the numbers of doctors receiving the email invitation to take part were unsuccessful.

### Implications for practice and research

The present study supports the theoretical consideration of attitudes, subjective norms and perceived behaviour control in predicting intentions to engage in professional behaviours. As proposed by others [4], the TPB could be adopted by medical researchers and educators as a unifying theoretical framework with which to guide professionalism education and its assessment. We encourage researchers, medical educators and organisations, including policymakers, to consider the variables of the TPB. First, such training should aim at empowering students/trainees and increasing beliefs in their capabilities. Perceived behaviour control is especially important when considering complex professional behaviours, such as raising concerns. Second, creating learning environments which foster positive attitudes towards professionalisms is essential. Third, the TPB highlights the importance of subjective norms and medical educators should not forget that they are role models who are influential in creating behavioural norms which determine their students/trainees future actions. The learning process is much more than just gaining new skills, it creates behavioural rules and expectations of our future doctors. For medical schools, this can mean addressing the hidden curricula [4, 42] and institutional norms: behaviours by teachers and others in clinical settings can either reinforce or undermine professional behaviours [43].

Another important advancement to the medical education field of this study is the development of a questionnaire that has the ability to predict future professional behaviours. Professionalism is challenging to assess [41, 44]. A literature review spanning 20 years found existing assessment methods of professionalism have predominately measured medical ethics [41] with many existing instruments not fully examined for reliability and validity [41, 45]. The questionnaire developed in this study measured three professional behaviours (reflective practice, raising concerns, and use of guidance) and had good internal consistency. The questionnaire was developed based on the TPB guidelines [33] which enables us to understand the factors which influence professional behaviours that could enhance professionalism-in-action and can also be used as a tool to evaluate interventions designed to change professional behaviours.

## Conclusions

The present study addressed the need for more theory-based research to understand clinicians’ behaviours [5–8] and investigated the utility of the TPB in investigating doctors’ professionalism. The findings revealed that more positive attitudes, stronger subjective norms and greater perceived behavioural control predicted doctors’ professional behaviours, e.g., intentions to raise concerns, engage in reflective practice, and use confidentiality guidance. These findings support the idea that researchers, medical educators and organisations should consider the variables of the TPB in their research and practice. Nevertheless, the observed variation in efficacy of prediction between the different professional behaviours might indicate that doctors have less control over certain behaviours (such as raising a concern) and therefore work environment and organisational factors are more influential.

## Supplementary information

**Additional file 1:** **Table S1.** Questionnaire description.

## Data Availability

In order to protect the confidentiality of research participants, the data generated and analysed during the current study are not publicly available.

## Abbreviations

ATT: Attitudes
BME: Black and Minority Ethnic
DoaD: Duties of a Doctor programme
EEA: European Economic Area
GMC: General Medical Council
HEE: Higher Education England
IMG: International Medical Graduates
INT: Intentions
LRMP: List of Registered Medical Practitioners
NHS: National Health Service
PBC: Perceived behavioural control
PMQ: Primary medical qualification
SN: Social norms
TPB: Theory of Planned Behaviour
TRA: Theory of Reasoned Action
UCL: University College London
UKGs: Non-UK graduates
UKGs: UK graduates
