# Supplementary material for: A theory-based study of doctors’ intentions to engage in professional behaviours
Source: BMC Med Educ. 2020 Feb 10;20:44. doi: 10.1186/s12909-020-1961-8 (PMC7011214; doi:10.1186/s12909-020-1961-8)
Supplement: Supplementary file 1 — Additional file 1: Table S1. Questionnaire description. [file 12909_2020_1961_MOESM1_ESM.docx]

# Additional file 1

Table S1. Questionnaire description

| **TPB theory constructs** | **Description of scales** | | | | | | | | | |
| --- | --- | --- | --- | --- | --- | --- | --- | --- | --- | --- |
|  | **Raising concerns** | | | **Reflective practice** | | | **The use of confidentiality guidance** | | |  |
|  | **No of items** | **Scoring** | **Example** | **No of items** | **Scoring** | **Example** | **No of items** | **Scoring** | **Example** |  |
| Attitudes | 3 | Bipolar scale (1 to 7) | *“Overall, I think that raising a concern is”:* difficult (1) - easy (7) | 6 | Bipolar scale (1 to 7) | *“Reflecting on my practice makes me a better doctor”* | 6 | Bipolar scale (1 to 7) | *“Overall, think that the GMC confidentiality guidance is hard to apply”* |  |
|  | 1 | Likert scale (1 – strongly disagree; 7 – strongly agree) |  | 2 | Likert scale (1 – strongly disagree; 7 – strongly agree) |  | 2 | Likert scale (1 – strongly disagree; 7 – strongly agree) |  |  |
| Subjective norms | 2 | Likert scale (1 – strongly disagree; 7 – strongly agree) | *“People who are important to me think I should not report a concern if I have one”* | 2 | Likert scale (1 – strongly disagree; 7 – strongly agree) | *“It is expected of me that I reflect on my practice”* | 2 | Likert scale (1 – strongly disagree; 7 – strongly agree) | *“How much pressure you feel from your trust to use the GMC’s confidentiality guidance”* |  |
|  | 9 | Likert scale (1 – no pressure; 7 – strong pressure) |  | 10 | Likert scale (1 – no pressure; 7 – strong pressure) |  | 9 | Likert scale (1 – no pressure; 7 – strong pressure) |  |  |
| Perceived behavioural control | 1 | Likert scale (1 – strongly disagree; 7 – strongly agree) | *“I am confident that I can raise a concern if I want to”* | 1 | Bipolar scale (1 – difficult; 7 - easy) | *“For me to reflect on my practice is difficult”* difficult (1) -easy (7) | 3 | Likert scale (1 – strongly disagree; 7 – strongly agree) | *“I have enough time to refer to the GMC confidentiality guidance”* |  |
|  | 1 | Bipolar scale (1 – difficult; 7 - easy) |  |  |  |  | 1 | Bipolar scale (1 – difficult; 7 - easy) |  |  |
| Intentions to change behaviour | 3 | Likert scale (1 – strongly disagree; 7 – strongly agree) | *“I plan to raise a concern if I have one in my workplace”* | 3 | Likert scale (1 – strongly disagree; 7 – strongly agree) | *“I intend to reflect on my practice”* | 3 | Likert scale (1 – strongly disagree; 7 – strongly agree) | *“I don’t plan to use the GMC confidentiality guidance” (reverse)* |  |
